# Effects of alkaloids on peripheral neuropathic pain: a review

**DOI:** 10.1186/s13020-020-00387-x

**Published:** 2020-10-02

**Authors:** Chunhao Zhu, Ning Liu, Miaomiao Tian, Lin Ma, Jiamei Yang, Xiaobing Lan, Hanxiang Ma, Jianguo Niu, Jianqiang Yu

**Affiliations:** 1 Department of Pharmacology, College of Pharmacy, Ningxia Medical University, No. 1160 Shengli Street, Yinchuan, 750004 Ningxia China; 2 Ningxia Collaborative Innovation Center of Regional Characteristic Traditional Chinese Medicine, Ningxia Medical University, No. 692 Shengli Street, Yinchuan, 750004 Ningxia China; 3 Department of Anesthesiology, General Hospital of Ningxia Medical University, No. 804 Shengli Street, Yinchuan, Ningxia Hui Autonomous Region, 750004 Ningxia China; 4 Ningxia Key Laboratory of Craniocerebral Diseases of Ningxia Hui Autonomous Region, Ningxia Medical University, No. 1160 Shengli Street, Yinchuan, 750004 Ningxia China

**Keywords:** Chinese herbal medicines, Alkaloids, Peripheral neuropathic pain, Analgesia

## Abstract

Neuropathic pain is a debilitating pathological pain condition with a great therapeutic challenge in clinical practice. Currently used analgesics produce deleterious side effects. Therefore, it is necessary to investigate alternative medicines for neuropathic pain. Chinese herbal medicines have been widely used in treating intractable pain. Compelling evidence revealed that the bioactive alkaloids of Chinese herbal medicines stand out in developing novel drugs for neuropathic pain due to multiple targets and satisfactory efficacy. In this review, we summarize the recent progress in the research of analgesic effects of 20 alkaloids components for peripheral neuropathic pain and highlight the potential underlying molecular mechanisms. We also point out the opportunities and challenges of the current studies and shed light on further in-depth pharmacological and toxicological studies of these bioactive alkaloids. In conclusion, the alkaloids hold broad prospects and have the potentials to be novel drugs for treating neuropathic pain. This review provides a theoretical basis for further applying some alkaloids in clinical trials and developing new drugs of neuropathic pain.

## Background

Neuropathic pain is a type of chronic pain directly caused by the injuries or dysfunction of the somatosensory nervous system [1], which further triggers anxiety and depression symptoms via worsening sleep, essential daily functioning, and quality of life of the patients. Chronic neuropathic pain has aroused a severe public health concern due to its heavy burden on families and society. The population prevalence of chronic neuropathic pain has been estimated to range from 6.9 to 10% [2–4]. It is noteworthy that the incidence of neuropathic pain is likely to escalate due to the improved survival rate of cancer patients, the aging population, and the aggressively growing incidence of diabetes mellitus.

Generally, neuropathic pain is further subdivided into central and peripheral neuropathic pain. Central neuropathic pain includes central lesions [5] and diseases (e.g., stroke [6], multiple sclerosis [7]), whereas peripheral nerve injuries or pathological changes induce peripheral neuropathic pain [8]. Besides, chemotherapy drugs- and diabetes-induced neuropathy are usually classified as peripheral neuropathic pain [9, 10]. So far, there has not been enough evidence that interventional management is safe and effective for neuropathic pain. Hence, Drug treatment remains a common route for pain relief. The first-line medication recommended by the International Association for the Study of Pain (IASP) includes pregabalin, gabapentin, and tricyclic antidepressants (TCAs), and topical application of lidocaine can relieve pain conditions in 30–50% of the patients [11–13]. However, some clinical overviews reported that review articles and guidelines tend to overstate gabapentin effectiveness [14]. More importantly, those agents generally are accompanied by serious side effects, such as cardiovascular events, sedation, and syncope [15]. A meta-analysis showed that pregabalin significantly increased the risks of adverse events (e.g., somnolence, dizziness, peripheral edema, visual disturbances, ataxia, and euphoria) [16]. Morphine, with the most satisfactory analgesic effect, shall be restricted in the routine clinical management of neuropathic pain due to its abuse risks and the shortcomings of analgesic tolerance [17, 18].

With the difficulties of finding new compounds and the safety-related drug recall, which makes the approval of new analgesic drugs more conservative, the global pharmaceutical industry is currently experiencing a new drug crisis of lacking of promising drug candidates, especially heavyweight drugs. Natural products remain important drug candidates in the development of novel medicines for neuropathic pain [19]. The increasing demand for alternative therapies, such as bioactive components with effective and safe antinociceptive properties in treating neuropathic pain, has been growing throughout the world [20–23]. The alkaloids derived from Chinese herbal medicines, as valuable sources of pharmaceutical products and leading compounds, have been of great significance in the research and development of anti-neuropathic pain drugs. More and more studies have demonstrated that low-dose alkaloids possess potential analgesic effects in various neuropathic pains models [24–30]. The present review focuses on the alkaloids, mainly quinolizidine alkaloids, isoquinoline alkaloids, indole alkaloids, diterpenoid alkaloids, and their analgesic effects on peripheral neuropathic pain.

## Alkaloids chemical structure, classification, and sources of Chinese herbal medicines

Alkaloids are the largest class of organic compounds containing nitrogen atom with the homophylic properties of alkali [31]. Many classic analgesics, such as morphine, codeine, and aspirin, are isolated from natural products. Therefore, the studies on the analgesic effect and mechanisms of active components and the discoveries of new analgesic drugs from plants provide grounds for innovative researches on analgesic drugs. Upon a literature survey, we identified 20 compounds with significant analgesic activities on peripheral neuropathic pain. The structures of these compounds are shown in Table 1. A compound may exist in various Chinese herbal medicines, and we have summarized all its sources (Table 2).

**Table 1:**
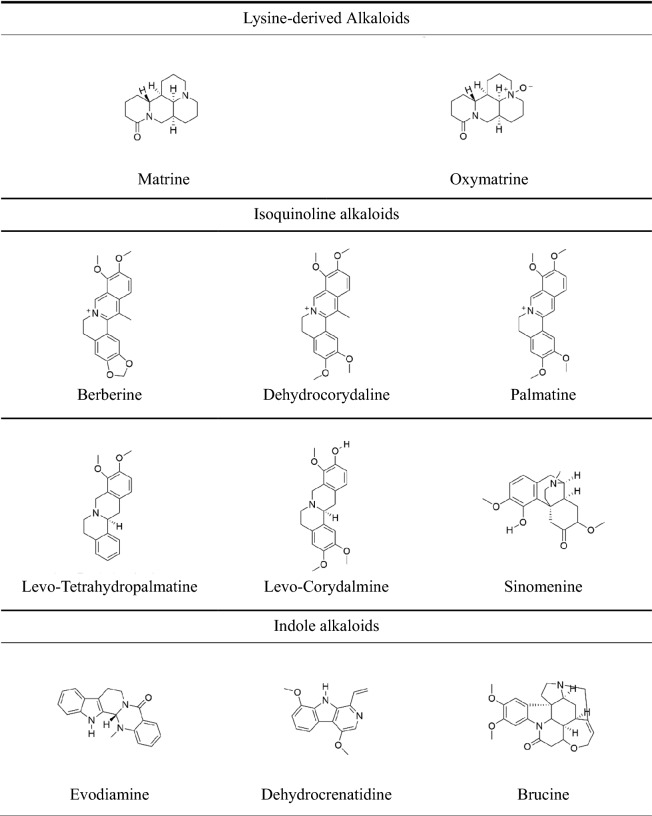

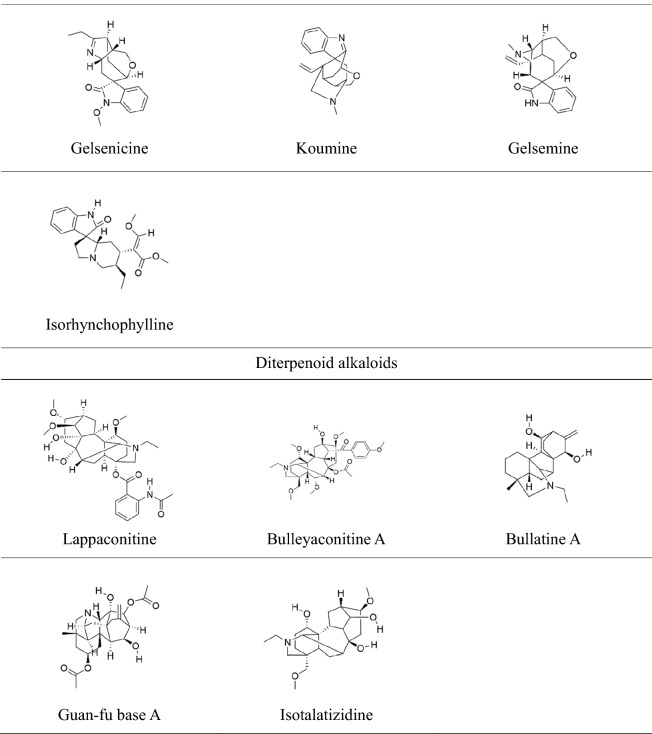
Chemical structures formula of different sub-groups of alkaloids

**Table 2:** Alkaloids with analgesic activity isolated from Chinese herbal medicines

Alkaloids Sub-groups	Example	CAS	Molecular formula	Sources	Latin name	References
Quinolizidine alkaloids	Matrine	519-02-8	C_15_H_24_N_2_O	Baicihua	*Sophora davidii*	[156]
				Kudouzi	*Sophora alopecuroides*	[156–158]
				Kushen	*Sophora flavescens*	[156, 159]
				Shandougen	*Sophora subprostrata*	[156]
	Oxymatrine	16837-52-8	C_15_H_24_N_2_O_2_	Baicihua	*Sophora davidii*	[156]
				Kudouzi	*Sophora alopecuroides*	[157, 158]
				Kushen	*Sophora flavescens*	[159]
				Shashenhuai	*Sophora moorcroftiana*	[156]
				Shandougen	*Sophora subprostrata*	[156]
Isoquinoline alkaloids	Berberine	2086-83-1	C_20_H_18_NO_4_^+^	Baiqucai	*Chelidonium majus*	[160]
				Baiyaozi	*Stephania cepharantha*	[160]
				Banruitangsongcao	*Thalictrum petaloideum*	[160]
				Huangbai	*Phellodendron amurense*	[161]
				Huanglian	*Coptis chinensis*	[162]
				Maweilian	*Thalictrum foliolosum*	[160]
				Yanguocao	*Thalictrum minus*	[160]
				Yanhusuo	*Corydalis yanhusuo*	[163]
	Levo-Tetrahydropalmatine	10097-84-4	C_21_H_25_NO_4_	Yanhusuo	*Corydalis yanhusuo*	[156]
				Xiatianwu	*Corydalis decumbens*	[156]
				Juhuahuanglian	*Corydalis pallida*	[156]
				Chibanyanhusuo	*Corydalis remota*	[164]
	Sinomenine	115-53-7	C_19_H_23_NO_4_	Bianfugegen	*Menispermum dauricum*	[160]
				Qingfengteng	*Sinomenium acutum*	[160, 165]
	Dehydrocorydaline	30045-16-0	C_22_H_24_NO_4_^+^	Yanhusuo	*Corydalis yanhusuo*	[160]
	Levo-Corydalmine	30413-84-4	C_20_H_23_NO_4_	Yanhusuo	*Corydalis yanhusuo*	[160]
	Palmatine	3486–67-7	C_21_H_22_NO_4_^+^	Banruitangsongcao	*Thalictrum petaloideum*	[164]
				Yanguocao	*Thalictrum minus*	[164]
				Huangbai	*Phellodendron amurense*	[164]
				Huanglian	*Coptis chinensis*	[164]
				Haisongzi	*Pinus koraiensis*	[164]
				Maweilian	*Thalictrum foliolosum*	[164]
				Xiatianwu	*Corydalis decumbens*	[164]
				Huanglian	*Coptis teetoides*	[164]
				Yanhusuo	*Corydalis yanhusuo*	[164]
				Alishanshidagonglao	*Mahonia oiwakensis*	[166]
				Qingniudan	*Tinospora sagittata*	[167]
Indole alkaloids	Evodiamine	518-17-2	C_19_H_17_N_3_O	Wuzhuyu	*Evodia rutaecarpa*	[168]
	Dehydrocrenatidine	65236-62-6	C_15_H_14_N_2_O_2_	Kumu	*Picrasma quassioides*	[101]
	Brucine	57-24-9	C_23_H_26_N_2_O_4_	Maqianzi	*Strychnos nux-vomica*	[169]
	Koumine	1358-76-5	C_20_H_22_N_2_O	Gouwen	*Gelsemium elegans*	[170]
	Gelsenicine	82354-38-9	C_19_H_22_N_2_O_3_	Gouwen	*Gelsemium elegans*	[170]
	Gelsemine	509-15-9	C_20_H_22_N_2_O_2_	Gouwen	*Gelsemium elegans*	[137, 170]
	Isorhynchophylline	6859-01-4	C_22_H_28_N_2_O_4_	Gouteng	*Uncaria rhynchophylla*	[171]
Diterpenoid alkaloids	Isotalatizidine	7633-68-3	C_23_H_37_NO_5_	Chuanwu	*Aconitum carmichaeli*	[172]
	Bulleyaconitine A	107668-79-1	C_35_H_49_NO_9_	Caowu	*Aconitum bulleyanum*	[173]
	Bullatine A	1354-84-3	C_21_H_31_NO_2_	Xueshangyizhihao	*Aconitum brachypodum*	[45]
	guan-fu base A	1394-48-5	C_24_H_31_NO_6_	Guanbaifu	*Aconitum coreanum*	[174]
	Lappaconitine	32854-75-4	C_32_H_44_N_2_O_8_	Ganwanwutou	*Aconitum finetianum*	[156]
				Gaowutou	*Aconitum sinomontanum*	[156]
				Niubian	*Aconitum barbatum*	[156]

## Effects of alkaloids on diabetic peripheral neuropathy (DPN)

Diabetic peripheral neuropathy (DPN) is one of the most common and refractory chronic complications of diabetes mellitus [32]. It is accompanied by burning, prickling, numbness, tingling sensation, allodynia, and hyperesthesia [33], which will affect 366 million individuals worldwide by 2030 [34]. Hyperglycemia mediated metabolic disorder is the primary pathogenesis for DPN. Hyperglycemia disturbs several metabolic pathways, such as advanced glycation end products (AGEs) [35, 36], hexosamine [37], polyol [38], protein kinase C (PKC) [39], and poly-ADP ribose polymerase (PARP) pathways [40] in the nervous system.

Koumine dose-dependently attenuated mechanical allodynia. The half-effective dose (ED_50_) of koumine (i.e., 0.063 mg/kg) is lower than the reported median lethal dose (LD_50_) of 99 mg/kg [27]. Berberine [41, 42] and palmatine [43] reduced streptozotocin (STZ)-induced mechanical allodynia and thermal hyperalgesia in a dose-dependent manner. Sinomenine [44] and Bullatine A [45] significantly upregulated the mechanical withdrawal threshold (MWT) and thermal withdrawal latency (TWL) of STZ mice.

### Neuroprotective effects of alkaloids on the sciatic nerve in DPN rodents

A study has reported that STZ could cause significant alterations in C-fibers and Aδ-fibers; alterations in these motor and sensory fibers caused a reduction in nociceptive threshold toward the mechanical and thermal receptors [46]. Also, peripheral nerve axon injuries and myelin degeneration can lead to abnormal sensory nerve conduction velocity (SNCV), which is an early characteristic of neuronal dysfunction in both diabetic neuropathies [47, 48]. SNCV directly reflects the change of axon caliber and myelin integrity induced by hyperglycemia [49], and establishes normal glycemia can be restored [47]. Berberine improved motor nerve conduction velocity (MNCV) and SNCV, which correlated with the upregulated expression of brain-derived neurotrophic factor (BDNF) and insulin-like growth factors I (IGF-I). It is reported that STZ administration caused significant downregulation in both IGF-I messenger ribonucleic acid (mRNA) and protein expression, whereas treatment with insulin significantly upregulated IGF-I mRNA in the sciatic nerve [42]. To sum up, the establishment of normal blood glucose is essential for the recovery of neurological function in diabetic mice, but berberine treatment of NP is not only through establishing normal blood glucose levels, which will be pointed out later. Interestingly, although koumine significantly improved SNCV and decreased myelinated nerve fibers' demyelination in the sciatic nerve, the blood glucose level of STZ-induced rats is not influenced [27]. The number and open characteristics of ion channels are likely to participate in which koumine restored SNCV in DPN mice [50].

### Regulation of alkaloids on oxidative stress and neuroinflammation in DPN rodents

**Fig. 1:**
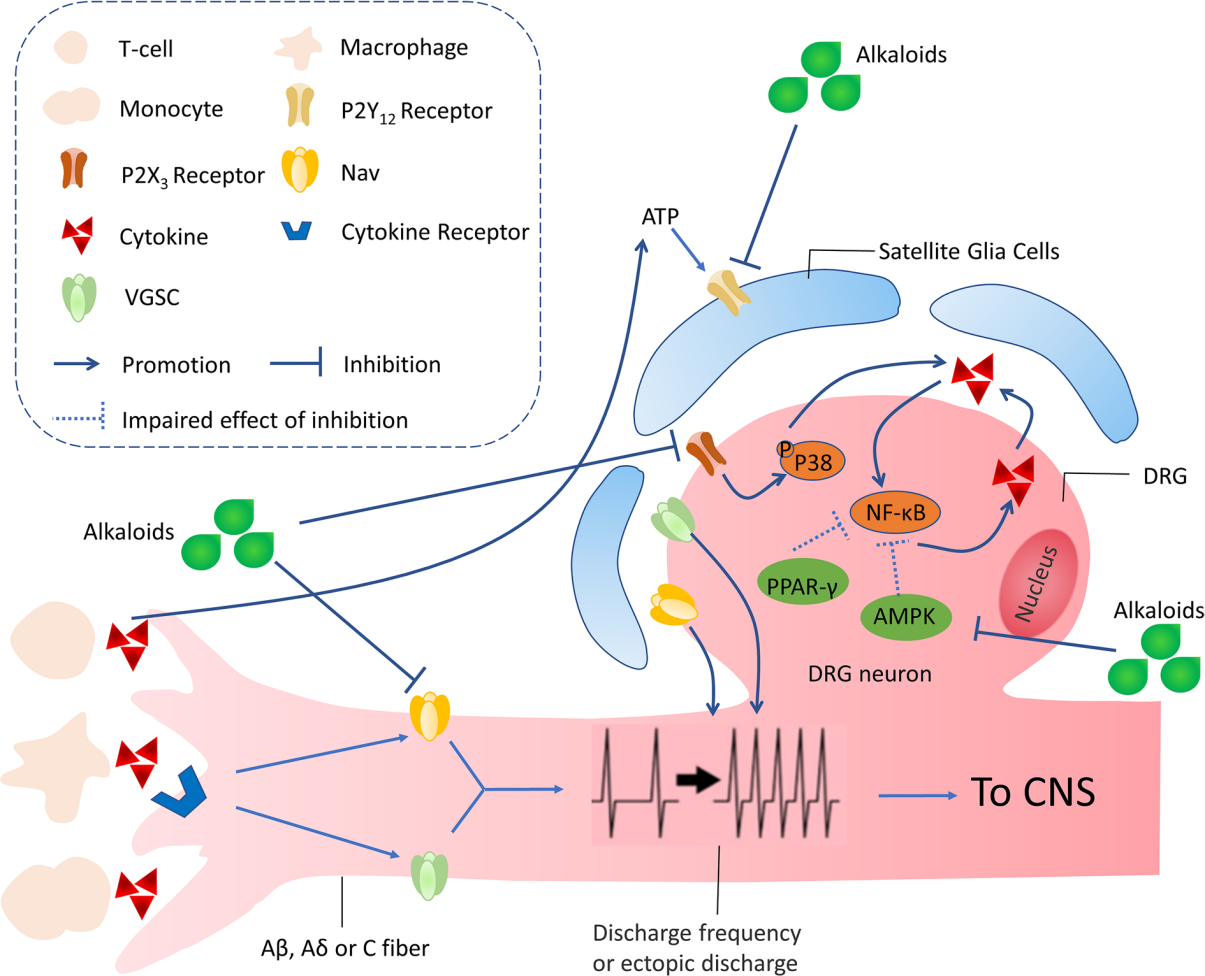
The main pharmacological mechanisms of alkaloids on treating DPN. Alkaloids alleviate DPN via inhibiting Nav channels-mediated ectopic discharge in afferent nerve fibers, suppressing purinergic signals in SGC and DRG neurons, and decreasing P38- and NF-κB-mediated peripheral neuroinflammation in DRG

Numerous researches have confirmed that hyperglycemia activates ROS that induces oxidative stress in the nervous system [51, 52]. The mechanism that contributes to increased oxidative stress includes alteration in PKC activity of, decreases Na^+^K^+^-ATPase activity, overproduction of prostaglandin, and accumulation of AGEs [53, 54]. The 40 mg/kg dose of berberine treatment significantly reversed STZ-induced oxidative stress and Na^+^K^+^-ATPase alteration. Berberine and palmatine are isoquinoline alkaloids stem from *Coptis chinensis*, which have been widely used to treat intestinal infection [55]. Liu et al. [41] recently revealed that berberine suppressed STZ-induced neuropathic pain via reducing satellite glial cells (SGCs) activation mediated neuroinflammation. The 20 and 40 mg/kg doses of berberine significantly decreased tumor necrosis factor-α (TNF-α), interleukin-1β (IL-1β), and interleukin-6 (IL-6) protein levels compared with STZ group rats. It is reported that the activation of adenosine 5′-monophosphate (AMP)-activated protein kinase (AMPK) and peroxisome proliferator-activated receptors-γ (PPAR-γ) can reduce the release of inflammatory cytokines [56, 57]. Treatment with berberine remarkably downregulated the protein expression of phosphatase 2Cα (PP2Cα) and upregulated the expression of Thr-172 protein(the site of AMPK phosphorylation) in dorsal root ganglia (DRG) as compared with STZ control rats [42] (Fig. 1). Substantial experimental evidence has supported that the activation of P2X receptors on glial cells are involved in the occurrence and maintenance of chronic pain by stimulating the production and release of TNF-α and IL-1β [58–61]. Besides, P2X_7_ receptors may be related to the comorbidity of DNP and depression [62]. Palmatine treatment ameliorated the comorbidity of DNP and depression via reducing the expression of TNF-α, IL-1β mRNA, and phosphorylation of extracellular regulates protein kinases 1/2(ERK1/2) protein in the hippocampus of DPN rats [43]. ATP-induced activation of P2X_3_ receptors might collaborate with the phosphorylation of P38 mitogen-activated protein kinase (P38MAPK) in DRG, causing mechanical and heat hyperalgesia [63]. Sinomenine alleviated high glucose-induced DPN and reduced the mRNA expression of P2X_3_ and the phosphorylation P38MAPK in DRG (Fig. 1). Furthermore, a molecular docking test revealed that sinomenine had an intense binding with P2X_3_ receptors [44, 64].

In conclusion, alkaloids attenuated diabetic neuropathy (Table 3) via restoring nerve function, inhibiting oxidative stress, and decreasing neuroinflammatory. In traditional Chinese medicine (TCM), *Coptis chinensis* have been employed to treat emaciation-thirst disease since ancient China. Berberine and palmatine are the main alkaloids in *Coptis chinensis*, which can treat many complications of STZ-induced diabetes [65, 66], including diabetic nephropathy [67]. As mentioned above, berberine and the other alkaloids had sound relieving effects on STZ-induced neuropathic pain and had a protective impact on sciatic nerves. Interestingly, berberine promoted axonal and myelin recovery by reversing the high glucose induced-downregulation of BDNF and IGF-I while restoring normal blood glucose levels in DM animals. In contrast, koumine exerted a neuroprotective effect by promoting neurosteroids in the sciatic nerve but did not affect the hyperglycemia level of diabetic animals. Similarly, a previous study that repeated koumine could significantly reduce the elevated total blood cholesterol levels in STZ-induced diabetic rats without affecting blood glucose. Thus, koumine could control cholesterol homeostasis, elevate neurosteroids levels and protect diabetic neuropathy by activating the liver X receptor [68], which may provide a potential novel therapeutic target for diabetic-induced NP. The potencies of koumine on mechanical allodynia, SNCV, and sciatic nerve morphology are different [27], which also suggests that koumine may exert anti-allodynic and neuroprotective effects by different mechanisms. The analgesic effect of berberine is a consequence of etiological treatment; in other words, berberine completely restored the hyperglycemia-induced neuropathic pain by hyperglycemia. Decreasing blood glucose can alleviate the signal activation induced by blood glucose and may be beneficial to the other complications caused by diabetes. Therefore, berberine is an excellent potential drug candidate for DPN. However, berberine should be applied at the appropriate phase of DPN, and the recovery of nerve function is most significant in the initial stage of DPN. In the future, it will be necessary to find out the hypoglycemic mechanism of berberine. Moreover, palmatine and koumine are also advantageous to diabetic-induced peripheral neuropathic pain.

**Table 3:** Effects of alkaloids on diabetic painful neuropathy (DPN)

Alkaloids	Animal/cell	Dose mg/kg (route of administration)	Effects/mechanisms of action				References
			Behavioral evaluation	Histopathological observation	Electrophysiology parameters	Biochemical/Molecular parameters/mRNA	
Koumine	Male Sprague–Dawley rats	0.056/0.28/1.4/7 mg/kg s.c 7 days	→ Blood glucose and body weight ↓Streptozotocin-induced mechanical allodynia	↑Myelin thickness, axon diameter and number of myelinated nerve fibers	↑SNCV	–	[27]
Berberine	Adult male and female Sprague–Dawley rats	10/20/40 mg/kg i.g 8 days	↓Streptozotocin-induced mechanical allodynia and thermal hyperalgesia	↓Necrosis, edema, inflammatory infiltration, and congestion after intraperitoneal administration of STZ in sciatic nerve ↓Split and disintegrated neurofilaments and swoln axonal mitochondria	↑MNCV and SNCV	Reverse Streptozotocin-induced oxido-nitrosative stress (SOD, GSH, MDA, and NO) and Na^+^K^+^-ATPase alteration ↑BDNF, IGF-1, PPAR-γ, IL-6, IL-1β and TNF-α	[42]
Berberine	Male C57BL/6 mice	The dose was not mentioned s.c 14 days	↓Streptozotocin-induced mechanical allodynia and thermal hyperalgesia	↓The relative Iba-1-positive area and GFAP-positive area reflected by the Iba-1 and GFAP fluorescence density after the treatment of berberine in spinal cord slices	–	↓The expression of TNF-α, IL-6, IL-1β, iNOS and COX-2 in spinal cord and DRG	[41]
Sinomenine	Male Sprague–Dawley rats HEK293 cells	40 mg/kg i.p 10 Μm(in vitro)	↓Streptozotocin -induced thermal hyperalgesia and mechanical hyperalgesia	–	↓ATP-activated current in HEK293 cells transfected with the pEGFP-hP2X3 plasmid	↓p-P38MAPK and P2X_3_ protein in DRG ↓P2X_3_ mRNA in DRG	[44, 64]
Palmatine	Male Sprague- Dawley rats	30 mg/kg i.p 14 days	↓Streptozotocin-induced mechanical hyperalgesia and thermal hyperalgesia ↓Depressive like behavior	↓Fluorescence intensity of co-localization of P2X_7_ and GFAP in the hippocampus ↓Positive signal of P2X_7_ protein in the hippocampus	–	↓P2X_7,_ TNF-α and IL-1β mRNA ↓p-ERK1/2 in the hippocampus	[43]
Bullatine A	Male adult Wistar rats	0.3/1/3/10/30 mg/kg s.c 0.3/1/3/10/30 μg i.t	↓Mechanical allodynia and thermal hyperalgesia	–	–	–	[45]

↑: Enhanced/Increased/Upregulate↓: Attenuate/Downregulate/Decrease/Suppress/Inhibit/Prevent

## Effects of alkaloids chemotherapy-induced peripheral neuropathy (CIPN)

CIPN is one of the most common complications caused by chemotherapy agents. The typical sensory impairment of CIPN includes numbness, paresthesias, evolving spontaneous hyperalgesia and allodynia to mechanical and thermal stimuli in extremities [69], and motor symptoms such as reduced balance control and distal weakness [70]. Generally, CIPN symptoms arise after repeating chemotherapy for three or four cycles of, mainly depend on the chemotherapeutic agent that is used and the cumulative dose of these drugs; however, the onset of CIPN symptoms has also been observed immediately in some patients after chemotherapy; these symptoms may become permanent and will continue for years [70, 71]. CIPN is suspected to be a complex phenomenon resulting from the interrelation of different mechanisms. It has been observed that anticancer drugs may cause neuronal damage in various ways, such as nuclear and mitochondrial DNA damages, ion channel disturbances (i.e., calcium, sodium, and potassium), impairment of axonal transport, and inflammatory process [72–74]. The effects of alkaloids on CIPN are shown in Table 4.

**Table 4:** Effects of alkaloids on chemotherapy-induced peripheral neuropathy (CIPN)

Alkaloids	Animal/cell	Dose mg/kg (route of administration)	Effects/mechanisms of action				References
			behavioral evaluation	histopathological observation	Electrophysiology parameters	Biochemical/Molecular parameters/mRNA	
Berberine	Male Wistar rats	10/20 mg/kg i.p 8 days	→ Paclitaxel-induced heat hyperalgesia and cold allodynia	↑Shrunken and swollen axons with myelin breakdown → Axon number	–	↓MDA and GPx levels in the sciatic nerve tissue ↑GSH and SOD levels and Nrf2 mRNA in the sciatic nerve tissue	[87]
Evodiamine	Male Sprague–Dawley rats	5 mg/kg i.p 3 days	↓Paclitaxel-induced mechanical and thermal hypersensitivity	↑IENFs	–	↓IL-1β, IL-6, TNF-α and MCP-1 ↑PGC-1α, UCP2 and SOD2 mRNA in DRG	[78]
Matrine	Male ICR mice	15/30/60 mg/kg i.p 11 days	↓Vincristine-induced mechanical allodynia, cold allodynia, heat hyperalgesia and mechanical hyperalgesia → Motor Coordination → SFI	↓The loss of myelinated nerve fibers, part of the axons arranged in irregular swelling, rise in the number of vacuoles	↑Vincristine-induced loss of SNCV and SNAP amplitudes in a dose dependent manner	↑T-AOC, SOD, GSH-Px and TCA activity ↓MDA and MPO	[82]
Bulleyaconitine A	Male Sprague–Dawley rats	0.1/0.4/0.8 mg/kg i.g	↓Mechanical allodynia and thermal hyperalgesia	–	↓HFS induced LTP ↓The frequency of sEPSCs and mEPSCs in laminar II neurons → The amplitude of sEPSCs and mEPSCs in laminar II neurons	–	[92]
*Corydalis saxicola* Bunting total alkaloids	Male Sprague–Dawley rats	30/60/120 mg/kg i.g	↓Cisplatin-induced mechanical allodynia, thermal hyperalgesia and cold Hyperalgesia	↓Cisplatin-induced vacuoles, neuron shrinkage, disordered satellite cells, and generally decreased Schwann cells	–	↓Cisplatin-induced pro-inflammation cytokines release in serum and foot supernatant of rats ↓ Cisplatin-induced p-p38 and TRPV1expression in DRG, TG, Spinal Cord and paw	[81]
Levo-corydalmine	Male ICR mice Primary astrocytes	5/10/20 mg/kg i.g 9 days 3/10/30 μM(in vitro)	Reversed vincristine-induced mechanical allodynia and thermal hyperalgesia	↓Fluorescence intensity of CXCL1 and p-NF-κB	–	↓TNF-α, IL-1β, CXCL1 and NF-κB activation in vivo and vitro	[89]

↑: Enhanced/Increased/Upregulate↓: Attenuate/Downregulate/Decrease/Suppress/Inhibit/Prevent

### Neuroprotective effects of alkaloids on intraepidermal nerve fibers and sciatic nerve in CIPN rodents

The loss of intraepidermal nerve fibers (IENFs) is primarily responsible for paclitaxel-induced neuropathic pain in rodent models [75, 76]. Moreover, prevention of the loss of IENFs can effectively ameliorate the sensory disturbance induced by paclitaxel [77]. Evodiamine ameliorated paclitaxel-induced peripheral neuropathy by attenuating IENFs injury [78]. All in all, evodiamine ameliorated paclitaxel-induced peripheral neuropathy by attenuating intraepidermal nerve fiber injuries. The mechanisms might involve the inhibition of peripheral neuroinflammation and activation of mitochondrial antioxidant functions. *Corydalis Saxicola* Bunting. Total Alkaloids (CSBTA) inhibit cisplatin-induced mechanical hyperalgesia, thermal hyperalgesia, and cold hyperalgesia. The significant loss of small-diameter myelinated or unmyelinated nerve fibers in the epidermis, including Aδ- and C-fibers, plays indispensable roles in cold allodynia, thermal hyperalgesia, and mechanical hyperalgesia in early CIPN [69, 77, 79, 80]. IENFs density is increased at 60 and 120 mg/kg doses of CSBTA, which result has been further confirmed by the histopathological experiments of DRG, revealing that CSBTA ameliorates cisplatin-induced vacuoles, neuron shrinkage, disordered satellite cells, and generally decreased Schwann cells [81].

The peripheral neuropathy model induced by chemotherapy agents is also accompanied by sciatic nerve function changes, such as SNCV and SNAP. However, our previous studies showed that chemotherapy drugs' application could not significantly reduce the sciatic nerve function index (SFI) of mice [82]. Matrine reversed vinorelbine-induced the decrease of SNCV and sensory nerve action potential (SNAP) amplitudes [82], and morphological evidence further support this notion.

### Regulation of alkaloids on oxidative stress in CIPN rodents

**Fig. 2:**
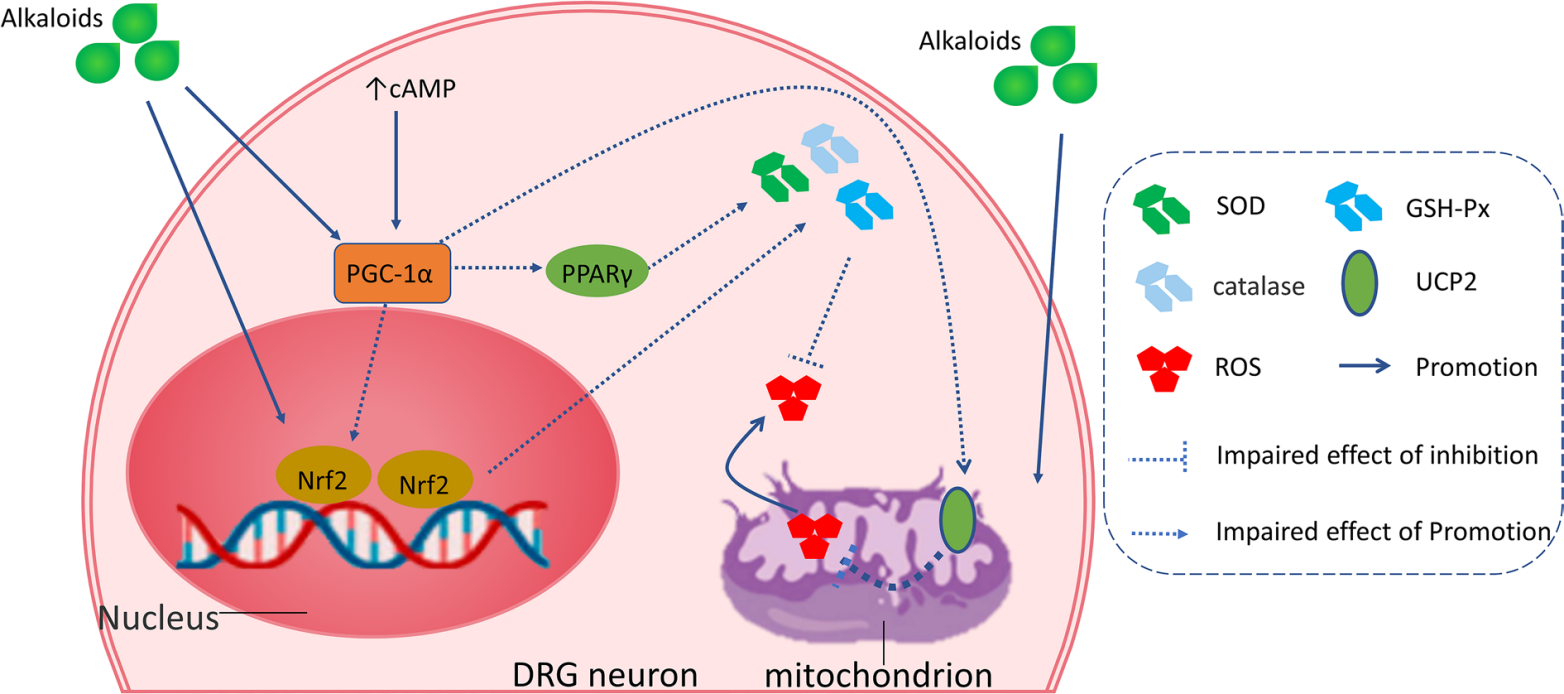
The main pharmacological mechanisms of alkaloids on treating CIPN. Alkaloids alleviate DPN via inhibiting oxidative stress and neuroinflammation in DRG

It must be considered that mammalian nerves are especially susceptible to free radicals, including reactive oxygen species (ROS) and reactive nitrogen species (RNS), due to their high content in phospholipids and axonal mitochondrion; besides, neuronal antioxidant defenses are weak [83]. Superoxide dismutase (SOD), catalase (CAT), and glutathione (GSH) are the major components of the antioxidant system, which maintains stability in DRG by scavenging excessive free radicals [84]. Uncoupling protein 2 (UCP2) on the mitochondrial inner membrane can reduce the electrochemical proton gradient and inhibit ROS generation [85]. Peroxisome proliferator-activated receptor-gamma coactivator 1-α (PGC-1α) can regulate the antioxidant system, and PGC-1 α can increase the level of antioxidant enzymes to protect neurons from the damages of ROS while decreasing glutathione in cells [86]. The treatment of evodiamine remarkably improved paclitaxel-induced mitochondrial dysfunction, evidenced by the restoration of PGC-1α, UCP2, and manganese superoxide dismutase (MnSOD). In vitro, studies found that evodiamine prevented the paclitaxel-induced loss of mitochondrial membrane potential and PGC-1α, UCP2, and MnSOD expression in DRG cells (Fig. 2) [78]. Singh et al. [87] found that berberine was effective in ameliorating the heat hyperalgesia and cold allodynia. Morphometric analysis of sciatic nerves revealed that berberine treatment significantly increased axon diameter and myelin thickness, but the axon number did not be increased compared with the paclitaxel group. Berberine prevented the increase in malondialdehyde level, the decreased GSH, and SOD in the sciatic nerve tissue. In oxidative stress, genes encoding antioxidant defending enzymes are activated, especially the nuclear erythroid 2-related factor 2 (Nrf2) gene, which increases the expression of SOD, glutathione peroxidase (GSH-Px), and catalase genes [88]. Berberine exerted analgesic and neuroprotective effects via upregulating Nrf2 mRNA and further enhancing antioxidant capacity in the sciatic nerve [87].

### Regulation of alkaloids on central and peripheral neuroinflammation in CIPN rodents

Administration of levo-corydalmine (L-CDL) combined with vincristine significantly reduced pain hypersensitivity and pro-inflammatory factors, such as TNF-α and IL-1β. In association with these changes, chemokine CXCL1 and its receptor CXCR2 were decreased by L-CDL in spinal astrocytes and neurons. Moreover, nuclear factor kappa-B (NF-κB) was involved in the production of CXCL1 in spinal astrocytes. In cultured astrocytes and primary neurons, CXCL1 was blocked by NF-κB small interfering RNA (siRNA) and was dose-dependently reduced by L-CDL, thus indirectly reducing the increase in CXCR2. (Fig. 2) [89]. Therefore, L-CDL has potential as an analgesic targeting the NF-κB-dependent CXCL1/CXCR2 signaling pathway to relieve vincristine-induced neuropathic pain. p38 is activated in DRG by peripheral inflammation and participates in the maintenance of heat hyperalgesia by regulating levels of the transient receptor potential channel family (TRPV1) [90, 91]. CSBTA also significantly reduced the expression of p38, p-p38, and TRPV1.

Repetitive administration of bulleyaconitine A (BAA) after treatment with paclitaxel produces a long-lasting inhibitory effect on thermal hyperalgesia, but not on mechanical allodynia. Consistently, the spinal synaptic transmission mediated by C-fibers but not by A-fibers is potentiated in paclitaxel-treated rats, and the effect is attenuated by either spinal or intravenous application of BAA. The frequency but not the amplitude of both spontaneous excitatory postsynaptic currents (sEPSCs) and miniature excitatory postsynaptic currents (mEPSCs) recorded in lamina II neurons was enhanced in paclitaxel-treated rats indicates that increase in presynaptic neurotransmitter release may contribute to the effect [92]. Furthermore, BAA could prevent and depress spinal LTP in both naive and paclitaxel-treated rats, but the inhibitory effect was more powerful in paclitaxel-treated animals than that in naïve animals. Treatment of BAA reduced the frequency of sEPSCs and mEPSCs in paclitaxel-treated rats but did not in naïve ones [92]. Taken together, BAA attenuated paclitaxel-induced neuropathic pain and depressed LTP at C-fiber synapses in the spinal dorsal horn, and inhibition of presynaptic transmitter release may be involved in the effect.

## Effects of alkaloids on Sciatic nerve chronic constriction injury (CCI) model

CCI-induced neuropathic pain includes several symptoms, such as spontaneous pain (tingling, burning, and electric-shock like), dysesthesia, paresthesias, allodynia, and hyperalgesia [93]. In addition, CCI results in intraneural edema, which produces a more significant reduction of Aβ-fibers, and a vast majority of Aδ-fibers are axotomized while large numbers of C-fibers are intact [94].

Table 5. summarized the effects of alkaloids in alleviating CCI-induced neuropathic pain. Alkaloids matrine [95] reduced among mechanical allodynia, cold allodynia, and thermal hyperalgesia, whereas guanfu base A [96] reduced mechanical and thermal hyperalgesia. Sinomenine [97], levo-tetrahydropalmatine [98], berberine [99], brucine [100], dehydrocrenatidine [101], koumine [102] and isotalatizidine [25] reduced mechanical allodynia. Besides, brucine, koumine, gelsenicine [26], and isorhynchophylline [103] inhibited CCI-induced thermal hyperalgesia. It is reported that matrine [95], levo-tetrahydropalmatine [98], berberine [99], and sinomenine [97] could not affect the ability of spontaneous activity and motor coordination of CCI mice. In other words, these alkaloids did not induce sedation in CCI mice. Sinomenine dose-dependently reversed the increased immobility time in rats receiving CCI but did not affect the duration of immobility in the forced swimming test in healthy animals, suggesting that sinomenine attenuated chronic pain-induced depressive-like behaviors.

**Table 5:** Effects of alkaloids on sciatic nerve chronic constriction injury (CCI)

Alkaloids	Animal/cell	Dose mg/kg (route of administration)	Effects/mechanisms of action				References
			Behavioral evaluation	Histopathological observation	Electrophysiology parameters	Biochemical/molecular parameters/mRNA	
Isotalatizidine	Female C57BL/6 mice BV2 microglial cells Primary microglial cells	0.1/0.3/1.0 mg/kg i.t 25 μM(in vitro)	↓Mechanical allodynia	↑Fluorescence intensity Dynorphin A in spinal cord	–	↑p-CREB, p-ERK1/2, dynorphin A in the spinal cord tissue ↑prodynorphin mRNA	[25]
Gelsenicine	Male ICR mice	0.8/4/20 mg/kg s.c	↓Thermal hypersensitivity	–	–	–	[26]
Dehydrocrenatidine	Male Sprague–Dawley rats Primary DRG neurons	50/150/250 µg/kg i.t	↓Mechanical allodynia	–	↓TTX-S and TTX-R currents in DRG	–	[101]
Matrine	Male ICR mice	7.5/15/30 mg/kg i.p 8 days	↓Mechanical allodynia, cold allodynia and thermal hyperalgesia → Motor coordination → Spontaneous locomotor	–	–	–	[95]
Guanfu Base A	Male Sprague Dawley rats HEK293 cells	40 mg/kg i.p 14 days	↓Mechanical and thermal hyperalgesia	↓Co-expression values of P2Y_12_ and GFAP	–	↓The expression of P2Y_12_ mRNA, P2Y_12_ protein, TNF-α and p-p38 MAPK in L4 –L6 DRGs ↓ADP-induced cAMP concentration in HEK293 cell	[96]
Sinomenine	Male Sprague–Dawley rats	10/20/40 mg/kg i.p 2 weeks	↓Mechanical allodynia ↓Depressive like behavior ↓Locomotor coordination	–	–	–	[97]
Levo-tetrahydropalmatine	Male ICR mice	20 nmol i.p 2 nmol i.t	↓Percentage withdrawal response frequency → Motor coordination	–	–	↓pNR1 in spinal dorsal horn	[98]
Berberine	Male Sprague–Dawley rats	5/10/20 mg/kg i.p	↓ Mechanical allodynia and cold allodynia Berberine does not induce sedation			↓MPO, MDA in Sciatic nerve	[99]
Brucine	Male C57BL/6 mice	10/30 mg/kg	↓Thermal hypersensitivity and mechanical allodynia	–	↓Action potential (AP) firing activity of DRG neurons ↓TTX-r and TTX-s currents	–	[100]
Isorhynchophylline	Adult maleC57BL/6 J mice	5/15/45 mg/kg i.g Twice per day for 14 days	↓Tactile allodynia and thermal → Motor coordination ↓Depression and anxiety like behavior	–	–	↑5-HT, NA → 5-HIAA, DA, DOPAC ↓5-HIAA/5-HT ratio, MAO-A	[103]
Oxymatrine	Male ICR mice	40/80/160 mg/kg i.p	↓Mechanical allodynia, cold allodynia and thermal hyperalgesia	↓ NR2B expression in the superficial dorsal horn	–	↓GAT-1 in spinal cord ↑GABAARα2	[109]
Oxymatrine	Male ICR mice	40/80/160 mg/kg i.p	↓Mechanical allodynia and thermal hyperalgesia	↑Immunostaining positive neurons of GABAAa2 ↓Immunostaining positive neurons of GAT-1	–	↓NR2B, p-ERK, p-CREB in spinal cord	[115]
Koumine	Male Sprague–Dawley rats Microglial BV2 Microglia	0.28/1.4/7 mg/kg s.c 7 days 25/50/100/200 μg/ml (in vitro)	↓Mechanical allodynia	↓ The fluorescence density of Iba-1, GFAP and TSPO expression in the spinal cord	–	↓IL-1β, TNF-α and TSPO in the spinal cord ↓ LPS-induced the protein levels and the mRNA expression of M1 markers (CD86, IL-6, IL-1β, and TNF-α) in BV2 cells	[102]
Koumine	Male Sprague–Dawley rats	0.28/1.4/7 mg/kg s.c twice per day for 7 days	↓Thermal hyperalgesia and mechanical allodynia	–	–	↑ Allopregnanolone → pregnenolone	[130]
Koumine	Male adult Sprague–Dawley rats	0.28/1.4/7 mg/kg s.c twice per day for 7 days	↓Hyperalgesia and allodynia	↑Fluorescence density of 3α‑HSOR in the ipsilateral dorsal horn of the lumbar spinal cord (L5–L6)	–	↑ The level of 3α‑HSOR mRNA and 3α‑HSOR catalytic activity in the spinal cord lumbar region L5–L6	[131]

↑: Enhanced/Increased/Upregulate↓: Attenuate/Downregulate/Decrease/Suppress/Inhibit/Prevent

### Regulation of alkaloids on excitatory and inhibitory synaptic transmission in CCI rodents

**Fig. 3:**
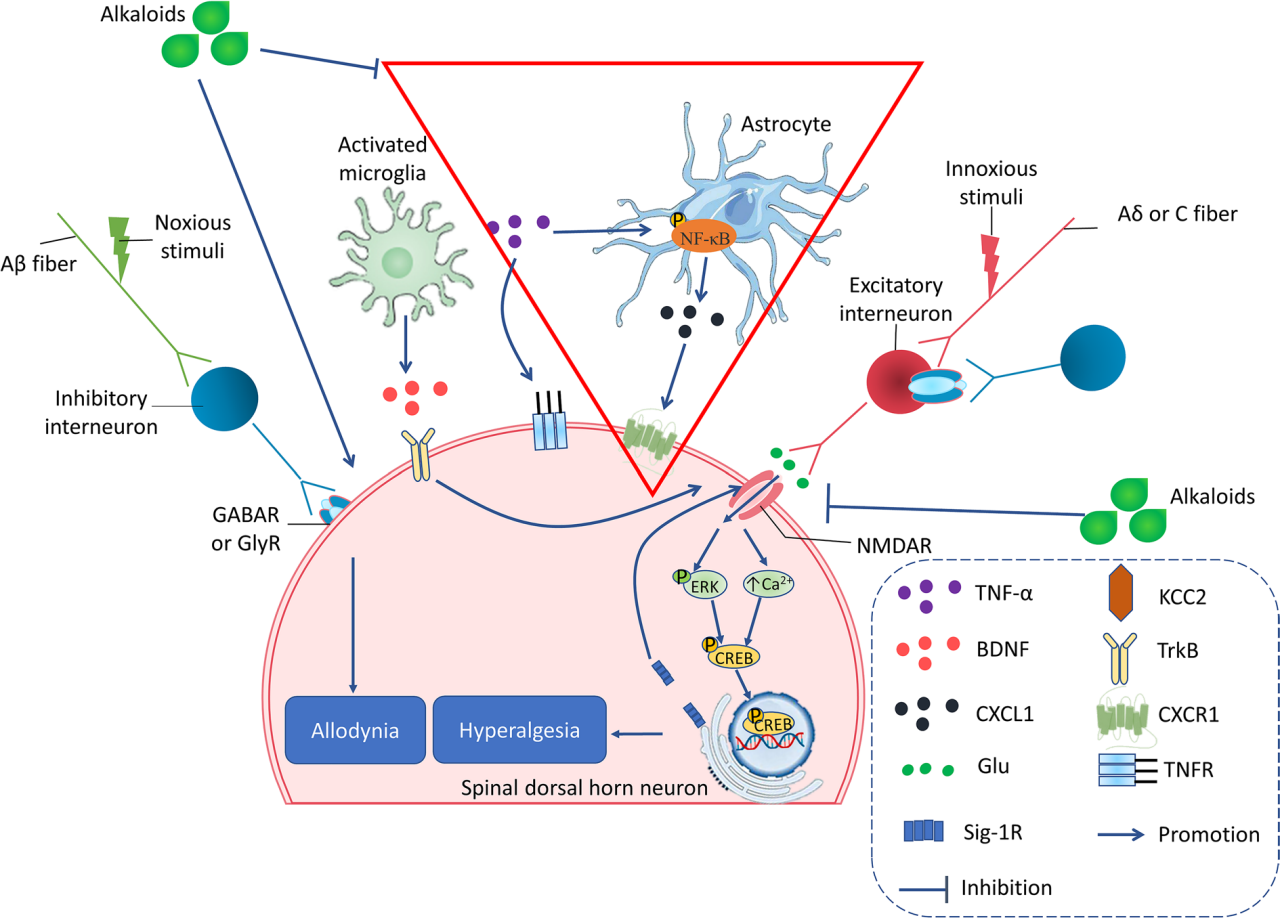
The main pharmacological mechanisms of alkaloids on treating CCI. Alkaloids attenuate mechanical allodynia in the CCI model via upregulating GABA receptor, inhibiting glia-mediated neuroinflammation, and inhibiting the activation of NMDAR

Following a nerve injury, both peripheral and central sensitization act as important pathogenesis of neuropathic pain, including sensitization and hyperexcitability of primary sensory neurons as well as the enhancement of excitatory synaptic transmission or the reduction of inhibitory synaptic transmission in the neurons of the central nervous system [104, 105]. The change of neurotransmitters (e.g., Glutamate and γ-aminobutyric acid (GABA)) within the SDH plays a vital role in the pathogenesis of chronic neuropathic pain [106]. In normal conditions, painful stimuli evoke action potentials in primary afferent neurons (Aδ- or C-fibers) and excite pain transmission in SDH neurons. SDH inhibitory interneurons containing GABA or glycine repress the excitatory interneurons that innervate SDH pain transmission neurons [107]. CCI decreases the inhibitory tone of GABA or glycine interneurons, which results in a relative enhancement of excitatory interneurons activity. The resulting hyperexcitability of pain transmission in neurons contributes to mechanical hypersensitivity. Chemically, although sinomenine is a morphinan analog, its antinociceptive effects are not abolished by an opioid receptors antagonist naloxone [108]. On the contrary, GABAA receptors antagonist bicuculine blocked the antinociceptive effects of sinomenine [97]. Taken together, these shreds of evidence reveal that sinomenine exerts significant antinociceptive effects on CCI-induced neuropathic pain via the GABAA-mediated mechanism. Previous studies in our laboratory found that oxymatrine [109] prevents the development of mechanical allodynia, thermal hyperalgesia, and cold allodynia in CCI mice by reversing CCI-induced the downregulation of GABAARα2 (Fig. 3). The spinal N-methyl-D-aspartate receptors (NMDARs) are known to contribute to the excitatory synaptic transmission within the spinal cord when they are evoked by nociceptive primary afferent stimuli, which plays important roles in the central sensitization of neuropathic pain [110]. Sigma-1 receptor (Sig-1R) is a unique transmembrane receptor that resides in the mitochondria-associated endoplasmic reticulum membrane (MAM) in the nervous system and has been found to play a pronociceptive role in neuropathic pain models [111, 112]. Sig-1R uncouples with immunoglobulin heavy chain binding protein (BiP) and translocates from the MAM to lipid rafts of the cell membrane when the concentration of Ca^2+^ decreases in MAM, and then modulates NMDAR responses (Fig. 2) [113, 114]. Previous studies have shown that levo-corydalmine alleviated vincristine-induced neuropathic pain. Levo-corydalmine and levo-tetrahydropalmatine are isoquinoline alkaloids isolated from Yanhusuo, levo-tetrahydropalmatine is an analog of levo-corydalmine (L-CDL) in which a methoxy group replaces the phenol hydroxyl group at the C_10_ position. The pretreatment of levo-tetrahydropalmatine (L-THP) remarkably suppressed mechanical allodynia and upregulated phosphorylation of NR1 in the spinal cord. Then, intrathecal treatment with L-THP combined with BD1047 (Sig-1R antagonist) synergistically reverse CCI-induced mechanical allodynia, indicating that L-THP alleviates CCI-induced neuropathic pain through modulating spinal Sig-1R activation (Fig. 3) [98]. It has been proposed that NMDA activation-induced Ca^2+^ influx can trigger an early phase of cAMP response element-binding protein (CREB) phosphorylation. However, delayed ERK signal cascade mediates a persistent phase of CREB phosphorylation, which is vital to the development and maintenance of chronic pain. Our study has shown that oxymatrine restores nerve injury-induced neuropathic pain, which could attribute to the inhibition of NR2B and ERK/CREB signaling pathway (Fig. 3) [115].

### Regulation of alkaloids on peripheral and central neuroinflammation in CCI rodents

In parallel to the changes in the activity of neurons, non-neurons cells, especially glial cells, are increasingly being recognized as essential contributors to the development and maintenance of neuropathic pain [116]. Microglia are activated in response to nerve injuries and then release pro-inflammatory cytokines such as TNF-α, IL-1β, and IL-6, thus initiating the neuropathic pain process [117]. Microglia are known to promote neuroinflammation not only by interacting with neurons but also by activating adjacent astrocytes [118]. The long-term subcutaneous administration of koumine decreased the fluorescence density of ionized calcium-binding adaptor molecule-1 (IBA-1) (microglia marker) and glial fibrillary acidic protein (astrocyte marker) in the ipsilateral spinal horn of CCI rats. Koumine downregulated the protein expression of IBA-1, Glial fibrillary acidic protein (GFAP), IL-6, IL-1β, and TNF-α by western blot analysis of spinal cord tissues [102]. Schwann cells and SGCs play fundamental and extensive roles in the primary treatment of nerve injuries in the peripheral nervous system. SGCs enwrap primary neurons in the DRG, where the P2Y_12_ receptor is highly expressed, which is involved in the nociceptive transmission. SGCs activation is characterized by increased expression of GFAP and the increased production of pro-inflammatory substances, such as ATP, ADP cytokines [119, 120], after which P2Y receptors are activated [8]. Guan-fu base A (GFA) decreases the expression of P2Y_12_ mRNA and protein in the DRG. The decreased co-expression of P2Y_12_ and GFAP is also observed in the DRG sections, which confirms the inhibitory effect of GFA on P2Y_12_ [96].

### Regulation of alkaloids on other pathological and physiological processes in CCI rodents

DRG modulates noxious stimuli and no-noxious stimuli from the peripheral nervous system through multiple ion channels expressed in DRG. Voltage-gated sodium channels (VGSC) contribute to generating the ectopic action potential in DRG neurons in various peripheral neuropathic pain conditions [121]. It should be noted that CCI-injured DRG neurons display altered expression levels of sodium channel subtypes, such as Nav1.3, Nav1.8, and Nav1.9 [122]. Dehydrocrenatidine (DHCT) is a β-carboline alkaloid from *Picrasma quassioides*, which suppress both tetrodotoxin-resistant (TTX-R) and sensitive (TTX-S) VGSC currents in DRG neurons with the half-maximal inhibitory concentration (IC_50_) values of 12.36 µM and 4.87 µM. Furthermore, DHCT prefers to interact with an inactivated state of VGSCs and prolongs the repriming time in both TTX-S and TTX-R VGSCs, transiting the channels into a slow inactivated state from a fast-inactivated state [101]. These data demonstrated that the analgesic effect of DHCT was possibly through inhibition of VGSCs. DHCT suppresses AP generation, suggesting that DHCT could inhibit the neuronal excitability. It shall be noted that various β-carboline alkaloids produce either a sedative, tremorgenic, anxiogenic, or convulsant effect by binding to benzodiazepine receptors acting as full, partial agonists, antagonists, or inverse agonists [123–127]. Whether DHCT interacts with benzodiazepine receptors and contributes to the neuronal excitability is currently unknown. The antinociceptive effect of brucine is related to the inhibition of TTX-R and TTX-S sodium channel directly, but the activation kinetics of Nav channels could not be changed [100].

Growing evidence suggests that endogenous neurosteroids are involved in the modulation of chronic pain. The endogenous biosynthesis of neurosteroids, such as allopregnanolone and pregnenolone, is upregulated in the spinal cord during neuropathic pain [128, 129]. Koumine increased allopregnanolone, but not pregnenolone in the spinal cord of CCI rats. The anti-neuropathic pain activity of koumine is mediated by further upregulation of allopregnanolone to an adequate level against neuropathic pain [130]. The decrease of allopregnanolone attributes to the fact that the key synthetase of allopregnanolone, 3α-hydroxysteroid oxidoreductase (3α-HSOR), is significantly upregulated by koumine [131]. It is worth mentioning that repeated subcutaneous administration of koumine is not associated with adverse effects commonly associated with opioids, such as physical and psychological dependence.

Single or consecutive administration of isorhynchophylline produces an analgesic effect lasting for 1 day or 3 days. Moreover, isorhynchophylline barely induce the change of motor function in the process of pain-related behavior tests. The analgesic effect is relatively rapid while reinitiating 10 days following interruption, indicating that the analgesic effect of isorhynchophylline seems to design neural plasticity changes. The descending monoaminergic projection is a vital pathway of endogenous pain modulation. Isorhynchophylline analgesia attributes to the escalated 5-hydroxytryptamine (5-HT) and the decreased 5-hydroxyindole acetic acid (5-HIAA)/5-HT ratio in the spinal cord. Pharmacological inhibition of spinal 5-HT corroborates that the escalated monoamine tone is responsible for the antinociceptive action of isorhynchophylline. Additionally, isorhynchophylline dose-dependently ameliorates the depressive and anxious conditions in neuropathic mice.

Activated MAPKs induce different intracellular signals and are also involved in maintaining neuropathic pain via regulating downstream cascade responses [132, 133]. Being consistent with this, MAPKs and microglial inhibitors remarkably attenuated neuropathic pain [134]. Shao et al. [25] found that isotalatizidine stimulated p38 and ERK1/2 in a cultured BV-2 cell line or primary microglia, which was completely inhibited by the respective inhibitors. Moreover, isotalatizidine induced phosphorylation of CREB is specifically mediated by the ERK1/2 pathway but not the p38 pathway. Interestingly, isotalatizidine induced the secretion of dynorphin A in the spinal cord tissue in CCI rat and ameliorated neuropathic pain, which can be reversed by the selective ERK1/2 inhibitor or selective CREB inhibitor. Therefore, the antinociception action of isotalatizidine in CCI-induced neuropathic pain was mediated via the activation of the ERK1/2/CREB/dynorphin A axis.

## Effects of alkaloids on sciatic nerve ligation (SNL) models

Table 6. summarized the effects of alkaloids in alleviating SNL-induced neuropathic pain. SNL-induced mechanical allodynia and thermal hyperalgesia have been reduced by lappaconitine, bullatine A (BA), bulleyaconitine A (BAA), gelsemine, koumine, and dehydrocorybulbine (DHCB). Lappaconitine antinociceptive activities are similar in mechanical allodynia and thermal hyperalgesia, with cumulative ED_50_ values of 1.1 mg/kg vs. 1.6 mg/kg and maximum effect (E_max_) values of 53 vs. 58% in SNL-induced neuropathic pain, respectively [29]. Moreover, subcutaneous administration of BA refreshed MWT and TWL in the cumulative dose range of 0.3 to 30 mg/kg, and dose–response analysis of BA at one hour after injection showed that E_max_ is 56.6% and 66.1% on MWT and TWL, respectively, and ED_50_ is 1.9 mg/kg and 0.7 mg/kg [135]. Both subcutaneous injection and intrathecal injection of BAA could effectively inhibit mechanical pain and thermal radiation pain in rats with E_max_ values of 60–100% and ED_50_ values of 42–59 μg/kg (s.c.), 94–126 ng (i.t.) [136]. Gelsemine [137] and koumine [130] can reverse SNL-induced mechanical allodynia and thermal hyperalgesia, and gelsemine at one hour after intrathecal injection exhibited its E_max_ of 51.2% and ED_50_ of 0.5 μg. DHCB can effectively reduce SNL-induced neuropathic pain at doses that do not induce sedation [138].

**Table 6:** Effects of alkaloids on sciatic nerve ligation (SNL) models

Alkaloids	Animal/cell	Dose mg/kg (route of administration)	Effects/mechanisms of action			Biochemical/molecular parameters/mRNA	References
			behavioral evaluation	histopathological observation	Electrophysiology parameters		
Lappaconitine	Male adult Wistar rats Primary neurons, astrocytes, and microglia	0.3/1/3/10 mg/kg s.c 0.3/1/3/10 μg i.t	↓Mechanical allodynia and thermal hyperalgesia	–	–	→The mRNA expression of dynorphin A, prodynorphin in microglia ↓LPS-induced the increase of TNF-α, IL-1β and IL-6 in microglia	[29]
koumine	Male adult Sprague–Dawley rats	0.28/1.4/7 mg/kg s.c Twice per day for 7 days	↓Mechanical allodynia and thermal hyperalgesia	–	–	–	[130]
Bulleyaconitine A	Male Sprague–Dawley rats	0.4 mg/kg i.g	↓Mechanical and thermal sensitivity	–	↓Morphine induced-the potentiation of spinal LTP at C-fiber synapses	↓p-PKCγ in SDH	[136]
Bullatine A	Male adult Swiss mice	0.1/0.3/1/3/10/30 mg/kg s.c	↓Mechanical allodynia and thermal hyperalgesia	–	–	–	[135]
Bulleyaconitine A	Male Sprague–Dawley rats Primary DRG neurons	–	↓Mechanical allodynia and thermal hyperalgesia	–	↓ Na^+^ channels in the uninjured neurons	–	[151]
Bullatine A	Male adult Wistar rats	0.3/1/3/10/30 mg/kg s.c 0.3/1/3/10/30 μg i.t	↓Mechanical allodynia and thermal hyperalgesia	↑Dynorphin A immunofluorescence staining in the ipsilateral SDH	–	→ LPS-induced the mRNA expression of TNF-α, IL-1β and IL-6 in microglia	[45]
Gelsemine	Male Wistar rats	0.03/0.1/0.3/1/3/10 μg i.t	↓Mechanical allodynia	–	–	↓The level of Gly α3 mRNA and protein	[137]
Dehydrocorybulbine	Male 129/sv mice	10 mg/kg i.p	↓Mechanical allodynia and thermal hyperalgesia	–	–	–	[138]

↑: Enhanced/Increased/Upregulate↓: Attenuate/Downregulate/Decrease/Suppress/Inhibit/Prevent

### Regulation of alkaloids on endogenous opioid peptides in SNL rodents

The analgesic effects of lappaconitine might attribute to the upregulation of dynorphin A in the central nervous system. The results support that the antinociceptive effects of lappaconitine are entirely blocked by intrathecal injection of the specific dynorphin A antibody and κ-opioid receptor antagonist [29]. Interestingly, it seems that the alkaloids bioactive components from the medicinal plants of *Ranunculus* can induce the production of dynorphin A in the spinal cord [25, 135, 139]. Huang et al. explored the analgesic mechanism of BA by studying the release of inflammatory factors and microglia dynorphin A. The results showed that BA could not inhibit the expression of inflammatory factors, including TNF-α, IL-1β, and IL-6 induced by SNL. However, it could significantly increase the expression and release of dynorphin A in the spinal cord and primary cultured microglia [45]. BAA's analgesic effects on neuropathic pain rats could be blocked by initial intrathecal injection of microglial activation inhibitor minocycline, dynorphin A antibody, and specific κ-opioid receptors antagonist nor-BNI. At the same time, BAA increased the expression of dynorphin A, which was inhibited by minocycline in primary cultured microglia (not neurons and astrocytes). Therefore, Li et al. believed that BAA's analgesic effect was produced by stimulating microglia in the spinal dorsal horn to release dynorphin A, which then acted on the κ-opioid receptors on the postsynaptic membrane [140].

**Fig. 4:**
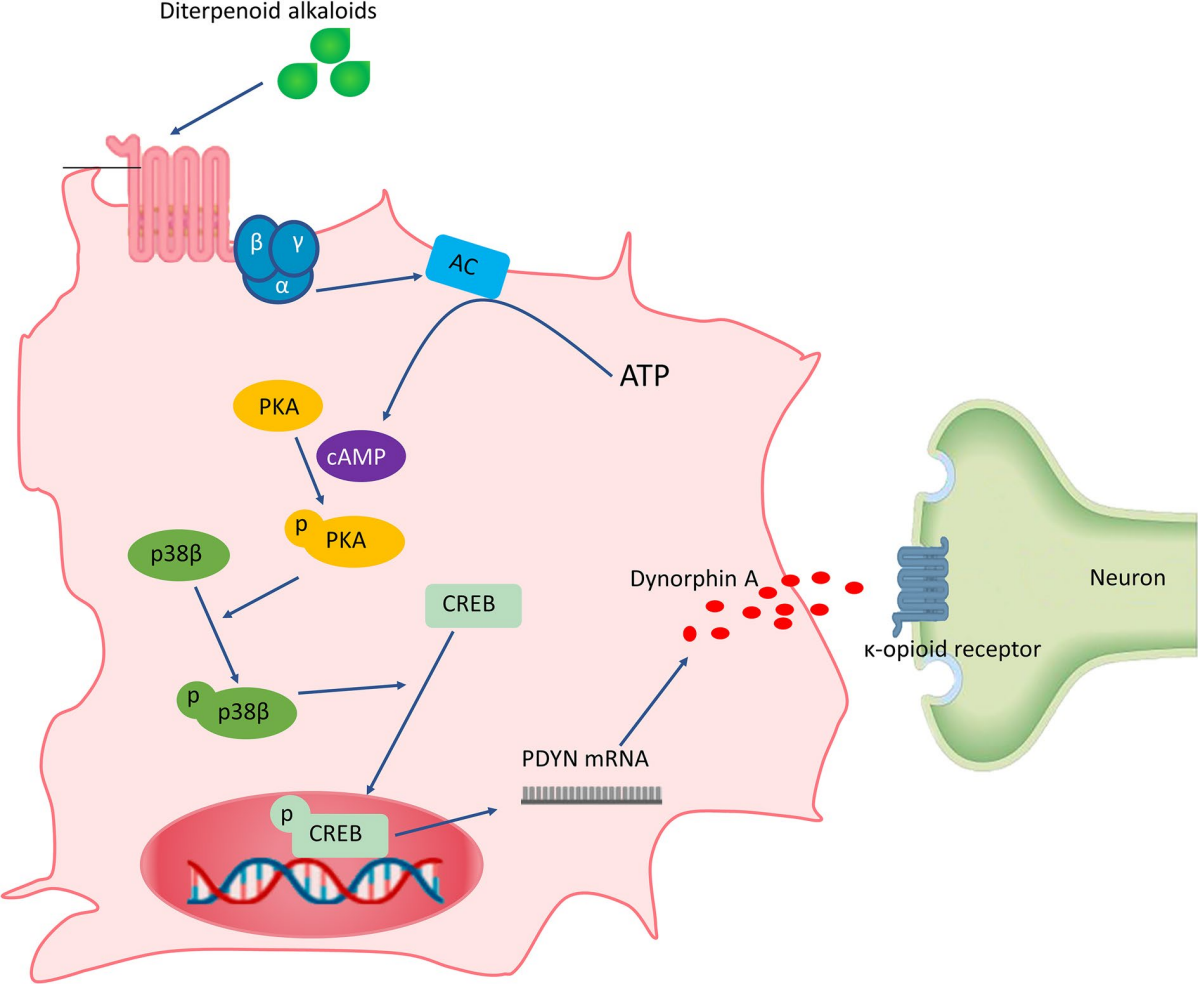
Proposed signal transduction pathways for diterpenoid alkaloids-induced analgesia on the SNL model. Following agonism of GPCRs, the cAMP/PKA, p38βMAPK, and CREB signals are successively activated, which mediate dynorphin A expression and subsequent antinociception

Comparing with the ED_50_ of these alkaloids (bulleyaconitine A > Lappaconitine = bullatine A) in neuropathic pain rats to EC_50_ of these diterpenoid alkaloids effects on promoting dynorphin A expression in primary microglia, we found that the analgesic activity is proportional to the effect of dynorphin expression, which further supports that the analgesic activity of these diterpenoid alkaloids is to promote dynorphin A expression. In addition, previous studies have also shown that aconitum extract has analgesic effects by affecting the release of dynorphin A in the spinal cord and acting on κ-opioid receptors [141–143]. Therefore, the molecular basis of diterpenoid alkaloids might stimulate the expression of dynorphin A in spinal cord microglia and further activate κ-opioid receptors on postsynaptic to produce an analgesic effect. The further study of Li et al. found that BAA can promote cAMP production, which could be blocked by G protein-coupled receptors (GPCRs) inhibitors. BAA can activate cAMP-dependent protein kinase A (PKA). PKA specifically upregulated p38 MAPK (not ERK MAPK or JNK MAPK) activity, thus promoting the phosphorylation of CREB and increasing the expression levels of prodynorphin and dynorphin A, further producing an analgesic effect [140]. It is worth noting that p38 activation mediates the release of inflammatory pain induced factors such as TNF-α, IL-1β, and IL-6 by microglia, but BAA also promoted the release of dynorphin A through p38 activation, which seems to be contradictory. In fact, p38 has four subtypes, including α, β, δ, and γ subtypes [144]. Furthermore, p38β siRNA could completely block the release of BAA-induced dynorphin A from microglia, but p38α siRNA did not affect it. These results suggested that p38β activation might be a common mechanism of opioid peptide released from microglia. Based on the above results, it is speculated that the analgesic molecular mechanism of lappaconitine, bullatine A (BA), and even other aconitine analogs are the same as that of BAA, and the expression of dynorphin A is mediated by activating the GPCRs/cAMP/PKA/p38β/CREB signaling pathway of microglia (Fig. 4) [140].

### Regulation of alkaloids on other pathological and physiological processes in SNL rodents

The specific antinociception of gelsemine in neuropathic pain was blocked by the glycine receptor antagonist strychnine with an apparent half-maximal inhibitory dose (ID_50_) value of 3.8 μg [170]. In addition, siRNA of glycine receptor α3 subunit was administered intrathecally for seven consecutive days, which completely blocked gelsemine induced-analgesia in neuropathic pain [170]. The results showed that gelsemine produced potent and specific antinociception in chronic neuropathic pain conditions without induction of apparent tolerance by activation of spinal glycine receptor α3 subunit [130]. LTP is abnormal electrical activity in neuropathic pain and morphine tolerance in the spinal cord. L5-SNL and continuous use of morphine can induce LTP lasting for 10 days in the spinal cord [145]. Previous studies have shown that activated protein kinase C-γ (PKCγ) is essential for LTP [146] and morphine tolerance [147] in neuropathic pain. Oral administration of BAA could relieve neuropathic pain and morphine-induced analgesic tolerance. Molecular biology and electrophysiology confirmed that BAA also inhibited LTP and the activation of PKCγ [136]. Interestingly, it has been shown that the spinal PKCγ expressing interneurons is activated only by innocuous inputs conducted by A-fibers [148]. The ectopic discharges mediated by the sodium channels in the primary afferent nerve [149] are significant for developing neuropathic pain [150]. The primary afferent fibers, especially A-fibers, discharge spontaneously following peripheral nerve injuries. BAA inhibited neuropathic pain by blocking the Nav1.7 and Nav1.3 in the peripheral nerve (Fig. 2) [151, 152]. These suggest that the inhibition of sodium channel in A-fibers may be responsible for the BAA-induced analgesia and LTP decrease. It has been found that BA could selectively antagonize P2X7 receptors, inhibit apoptosis of microglia induced by ATP, and inflammatory response mediated by P2X7 receptors [153].

## Discussion and conclusion

Most alkaloids are isolated from Chinese herbal medicines. The analgesic activities of various alkaloids are inextricably linked with the traditional application of Chinese herbal medicines. Yanhusuo is a kind of analgesic that is highly praised by Chinese medicine experts. It has the effects of promoting blood circulation, removing blood stasis, regulating qi, and relieving pain. According to the compendium of Materia Medica, Yanhusuo is specially used to treat all kinds of pains in the body. The main active components of Yanhusuo include dehydrocorydaline, levo-Tetrahydropalmatine, and levo-Corydalmine; these alkaloids have shown analgesic effects on neuropathic pain. Besides, *Aconitum*, such as *Aconitum carmichaelii*, *Aconitum kusnezoffii*, and *Aconitum sinomontanum*, mainly treat rheumatic arthralgia, acute and chronic pain. C18, C19, C20 diterpenoid alkaloids are the main active components of *Aconitum* and have significant analgesic effects. Previously studies indicated that bulleyaconitine A, lappaconitine, bullatine A, and isotalatizidine have good analgesic effects on neuropathic pain. However, Gouteng had a significant sedative effect in clinical application [154]. Therefore, in the study of alkaloids on neuropathic pain, we should pay more attention to whether alkaloids have sedative effects to avoid the temporary disappearance of pain-related behavior due to central inhibition. Compared to traditional single-target drugs, bioactive natural ingredients derived from herbs may provide additional benefits in preventing chronic neuropathic pain with improved efficacy and lower toxicity, and they represent an important source of drug discovery. In addition, it is a feasible method for researchers to search effective candidate compounds for the treatment of neuropathic pain by starting from the clinical application of traditional Chinese herbal medicines, and it is easier to identify the analgesic effects, toxic effects, and related mechanism of alkaloids.

Although previous studies have reported the pharmacological effects and mechanisms of action of the alkaloids isolated from Chinese herbal medicines, there is no review focusing on bioactive alkaloids in the treatment of peripheral neuropathic pain. Alkaloids inhibited peripheral and central neuroinflammation, reduced oxidative stress damage, and further ameliorated peripheral neuropathic pain. The expression of regulatory factors, including PGC-1α, PPAR-γ, and UCP2, the activation of the Nrf2, and the downstream upregulation of antioxidant enzymes, including SOD, GST, and GPx, have been proposed as common mechanisms underlying the antioxidant effects of these alkaloids. Moreover, most of the studies have suggested that the reversal of oxidative stress plays a critical role in the anti-inflammation effects of these alkaloids. Importantly, alkaloids' mechanism on neuropathic pain is not entirely inconsistent due to the pathological mechanism of different types of neuropathic pain that is different. However, the specific and detailed differences are still unclear. The influence of hyperglycemia and chemotherapy agents on peripheral nerves (e.g., sciatic nerve, epidermal nerve fibers) is serious due to a large number of mitochondria are susceptible to oxygen free radicals in the mammalian nervous system. Therefore, besides removing the etiology, it is necessary to improve the function of mitochondria in the nervous system for the treatment of DPN and CIPN. In addition, alkaloids exert analgesic effects on CCI-induced neuropathic pain via inhibiting the activation of microglia and astrocyte, regulating excitatory and inhibitory synaptic transmission, and regulating endogenous neurosteroids. Moreover, The analgesic mechanism of diterpenoid alkaloids on SNL-induced neuropathic pain is primarily to promote the release of endogenous opioid peptides. Fortunately, bioactive alkaloids have the characteristic of multi-target action on neuropathic pain. Specifically, koumine and berberine exerted analgesic effects on DPN and CCI rodents through different mechanisms. Based on clarifying the pathological mechanism of various neuropathic pain models, it is still necessary to further study the analgesic mechanisms of different alkaloids.

Among the alkaloids mentioned above, diterpenoid alkaloids are the most studied bioactive ingredients but their toxicity is a cause for concern. Researchers generally believe that diterpenoid alkaloids have the most severe toxic effects, such as cardiotoxicity and neurotoxicity, mediated by sodium channels. However, in the previous studies, no similar adverse reactions were found under the treatment dose, and the treatment index of BAA is about seven times that of aconitine. A wider therapeutic index means that the toxic effects and analgesic effects can be separated. Some studies have shown that sodium channel blockers could not reverse the analgesic effects of BAA. These researches further indicated that the analgesic and toxic effects of BAA were separated. In addition, diterpenoid alkaloids can promote the release of dynorphin A from spinal microglia and further exert analgesic effects with synaptic κ-opioid receptors. Direct agonists of κ-opioid receptors, especially synthetic non-peptide agonists, can cause severe irritability and side effects similar to psychosis. Because endogenous dynorphin A produced by diterpenoid alkaloids has a higher potency than exogenous dynorphin A, these alkaloids have no obvious adverse reactions, such as sedation, analgesia tolerance, under the effective treatment doses. In addition, the investigation of the structure–activity relationship is a meaningful way to balance the analgesic effects and toxic effects of alkaloids. At present, the structure–activity relationship of diterpenoid alkaloids has been studied. The following groups for the analgesic effects of C18 and C19 diterpenoid alkaloids are essential: a ring of trivalent N, acetyloxy or ethoxy substitution at the C_8_ position, aryl substitution at C_14_ position, and saturation of D ring. In addition, a 3α-hydroxyl group or 5-hydroxy group can enhance the analgesic activity of these compounds [155]. The ester groups at C_4_, C_8_, and C_14_ positions are active groups, as well as toxic groups. However, the introduction of ester groups at the C_3_ position, can improve the analgesic index of these alkaloids, which may separate the pharmacological activity from the toxicity. Therefore, it is possible to introduce the ester group at the C_3_ position while hydrolyzing the ester groups at C_4_, C_8_, and C_14_ positions to preserve the effectiveness and reduce toxicity.

In summary, even after taking the above-mentioned obstacles and concerns into account, the alkaloids are valuable drug candidates for treating neuropathic pain. Therefore, there is an urgent need to investigate their pharmacokinetic properties and establish the dose-time-pharmacology-toxicology relationships of alkaloids isolated from Chinese herbal medicines. Further preclinical research and clinical trials are crucial to demonstrate alkaloids' efficacy in alternative analgesia for neuropathic pain. Therefore, future research studies must address the anti-neuropathic pain targets and molecular mechanisms of Chinese herbal medicines and their monomeric compounds, combining laboratory anti-neuropathic pain researches with clinical practices, testing the reliability of Chinese herbal medicines against neuropathic pain, and promoting their application in practical neuropathic pain treatment.

## Data Availability

The datasets used and analyzed during the current study are available from the corresponding author on reasonable request.

## Abbreviations

TCAs: Tricyclic antidepressants
DPN: Diabetic peripheral neuropathic pain
AGEs: Advanced glycation end products
PKC: Protein kinase C
PARP: Poly-ADP ribose polymerase
ED_50_: Half-effective dose
LD_50_: Median lethal dose
STZ: Streptozotocin
MWT: Mechanical paw withdrawal threshold
TWL: Thermal paw withdrawal latency
SNCV: Sensory nerve conduction velocity
MNCV: Motor nerve conduction velocity
BDNF: Brain-derived neurotrophic factor
IGF-I: Insulin-like growth factors I
mRNA: Messenger ribonucleic acid
ROS: Reactive oxygen species
SGCs: Satellite glial cells
TNF-α: Tumor necrosis factor-α
IL-1β: Interleukin-1β
IL-6: Interleukin-6
AMPK: Adenosine 5′-monophosphate (AMP)-activated protein kinase
PPAR-γ: Peroxisome proliferator-activated receptors-γ
PP2Cα: Protein phosphatase 2Cα
DRG: Dorsal root ganglia
ERK1/2: Regulates protein kinases 1/2
P38MAPK: P38 mitogen-activated protein kinase
TCM: Traditional Chinese medicine
CIPN: Chemotherapy-induced peripheral neuropathy
IENFs: Intraepidermal nerve fibers
CSBTA: Corydalis Saxicola Bunting. Total Alkaloids
SFI: Sciatic nerve function index
SNAP: Sensory nerve action potential
RNS: Nitrogen reactive species
SOD: Superoxide dismutase
CAT: Catalase
GSH: Glutathione
UCP2: Uncoupling protein 2
PGC-1α: Peroxisome proliferator-activated receptor-gamma coactivator 1-α
MnSOD: Manganese superoxide dismutase
Nrf2: Nuclear erythroid 2-related factor 2
GSH-Px: Glutathione peroxidase
L-CDL: Levo-corydalmine
NF-κB: Nuclear factor kappa-B
siRNA: Small interfering RNA
TRPV1: Transient receptor potential channel family
BAA: Bulleyaconitine A
sEPSCs: Spontaneous excitatory postsynaptic currents
mEPSCs: Miniature excitatory postsynaptic currents
LTP: Long-term potentiation
CCI: Chronic constriction injury
GABA: γ-Aminobutyric acid
NMDARs: N-Methyl-D-aspartate receptors
Sig-1R: Sigma-1 receptor
MAM: Mitochondria-associated endoplasmic reticulum membrane
BiP: Immunoglobulin heavy chain binding protein
L-THP: Levo-tetrahydropalmatine
CREB: CAMP-response element binding protein
IBA-1: Ionized calcium-binding adaptor molecule-1
GFAP: Glial fibrillary acidic protein
LPS: Lpopolysaccharide
GFA: Guan-fu base A
VGSC: Voltage-gated sodium channels
DHCT: Dehydrocrenatidine
TTX-R: Tetrodotoxin-resistant
TTX-S: Tetrodotoxin-sensitive
IC_50_: Half maximal inhibitory concentration
3α-HSOR: 3α-Hydroxysteroid oxidoreductase
5-HT: 5-Hydroxytryptamine
5-HIAA: 5-Hydroxyindole acetic acid
SNL: Spinal nerve ligation
BA: Bullatine A
DHCB: Dehydrocorybulbine
E_max_: Maximum effect
ID_50_: Half-maximal inhibitory dose
PKCγ: Protein kinase C-γ
s.c.: Subcutaneous
i.g.: Intragastric
i.p.: Intraperitoneal
i.t.: Intrathecal
